# Adoption of health technologies for effective health information system: Need of the hour for Pakistan

**DOI:** 10.1371/journal.pone.0258081

**Published:** 2021-10-07

**Authors:** Madeeha Malik, Ahmad Furqan Kazi, Azhar Hussain

**Affiliations:** 1 Hamdard Institute of Pharmaceutical Sciences, Hamdard University Islamabad, Islamabad, Pakistan; 2 Dean Faculty of Pharmacy, Hamdard University Islamabad, Islamabad, Pakistan; DePaul University, UNITED STATES

## Abstract

Health information technology systems have the capacity to improve health outcomes for the patients thus ensuring quality and efficient services. Health information systems (HIS) are important tools in guidance towards patient safety and better outcomes. However, still, morbidity and mortality attributed to medical errors remain an important issue that needs to be addressed. The objective of the present study was to assess the health information system in terms of technological, environmental, organizational and human factors affecting the adoption as well as the perceptions of stakeholders along with barriers and constraints related to successful implementation. A descriptive cross-sectional study design was used. Prospective data was collected from primary sources by self-administering the pre-validated questionnaires as well as by physical verification of the availability of equipment. After data collection, data was analyzed to assess the health information management systems. The results of the present study showed that the health information system in Pakistan is not up to the mark. The equipment was mostly unavailable at the primary healthcare facilities. The staff was also unsatisfied with the available services. Administrative, financial and human constraints were identified as the major barriers towards successful implementation and management of HIS. The present study concluded that the health information system of Pakistan needs to be revamped. Health information management system partially existed at district and sub-district offices, while was completely absent at tertiary, secondary and primary healthcare levels. The poor adoption of health information technology systems at healthcare facilities might largely be attributed to insufficient human resources with limited resources and budget allocation for health in Pakistan. Effective and timely strategies involving all important stakeholders and healthcare professionals must be designed and implemented at the National level to restructure an affordable, resilient and quality healthcare system.

## Introduction

The healthcare sector is perhaps one of the largest and rapidly growing industries in the world with a market value of 7.6 trillion USD (EIU, 2016). As per an estimate, by 2020 the global spending on healthcare is expected to reach 8.7 trillion USD, possibly due to the improved integrated healthcare services that has increased the cost and life expectancy [1]. Health information systems must ensure safe and effective medication use, improved health outcomes and quality of services at a reduced cost [2]. Modern health information systems provide a comprehensive specialized integrated framework for managing different roles in the healthcare system including administrative, managerial, financial and clinical decision support systems [3]. Accurate and reliable information provides the basis for decision-making across all the segments of the healthcare system [4]. The use of these health information systems in hospitals can play an important role in decreasing medical errors [5].

Health information technology systems can improve the health outcomes for the patient resulting in provision of quality and efficient healthcare services [6]. Health information technologies are considered important tools for stakeholders in guiding them regarding patient safety and better outcomes. However, the financial and organizational barriers in their adoption are considered significant for implementation [7]. Various studies in the past have highlighted the benefits associated with the successful implementation of health information systems including; accessibility of patient data from health information management system, less time for data retrieval, readability & accuracy of data and reduction in medication errors [8–12]. However, considerable evidence also highlights multiple issues linked to the management of health information technologies including; high initial capital cost, technological and downtime issues, inadequately skilled & trained personnel as well as confidentiality and security issues related to health information management systems [13–15].

The healthcare system, even in the developed countries had undergone a paradigm shift and gradual improvement over the past two decades. A report published by the Institute of Medicine (USA) in 1999 shook the nation to its core and helped in raising awareness about deaths associated with medical errors. Around 44,000 people and as many as 98,000 people die every year in US hospitals as a result of preventable medical errors [16]. Most of the governments spent very little of their gross domestic product (GDP) on health till the twentieth century. US government’s expenditure on healthcare did not rise above 1% of GDP till the 1960s and 3% till 1980 [17]. Later in 1965, President Johnson introduced healthcare reforms through a joint session at Congress “The Great Society Legislation.” The act focused on the healthcare reforms for the older Americans (Medicare) as well for the betterment of the poor (Medicaid). Consequently, the spending on healthcare breached 2% of the GDP in 1970 and it kept on rising till, by 2009, a target of 7% of GDP was achieved [18].

Later in 2009, “The Health Information Technology for Economic and Clinical Health—HITEC Act” was introduced. The primary focus was to trigger the adoption of electronic health and medical records coupled with supporting health information technologies within the United States [19].

Major barriers identified in successful adoption include lack of capital, maintenance related costs, attitudes of healthcare professionals, unavailability of staff and countless return on investment [20]. Besides this, technological, environmental, organizational and human factors were also identified as the major barriers to successful adoption [21, 22]. Three primary challenges to prosperous adoption including; institutional challenges, human & social influence and challenges related to technology were highlighted by a study conducted in Malaysia [23]. Besides this, factors related to behavior and attitudes towards acceptance and use of technology had also been reported [24].

Attitude, lack of training and skills, policies and little administrative support were highlighted as the major barriers towards successful adoption in Indonesia [25]. Lack of technology was identified as the primary influential factor that decreased acceptance levels of health information technologies in Saudi Arabia [3].

The adoption of health information management systems in developing countries is perhaps worse than the developed ones. A study found the major influential factor that decreased acceptance level towards the health information system in Saudi Arabia was the unavailability of computers particularly laptops [3]. Another study conducted in Saudi Arabia highlighted that hospital size had a linear relation with the adoption of health system technologies [26]. Moreover, a study conducted in Malaysia reported that only 15.2% of the public hospitals had implemented the electronic health reporting system [21]. However, to counter such low levels of adoption, the Malaysian Government had taken various initiatives in the past. From ‘Seventh Malaysian Plan in 1996’ to ‘10th Plan in 2010’, the government had not only been making efforts to recognize the role of information and communication technologies but also had appreciated the use of such technologies in the health systems. The plan proposed to build 33 complete paperless hospitals in the public sector [27]. Nevertheless, a study reported that only 21 out of 138 public sector hospitals in Malaysia had implemented the electronic medical recording and reporting system either in one department or in all units [21]. A few East Asian countries like Singapore, Hong-Kong and Taiwan were found relatively more active in the adoption of health information technologies in comparison to countries having high adoption rates like Canada, England and Australia [28].

**Fig 1:**
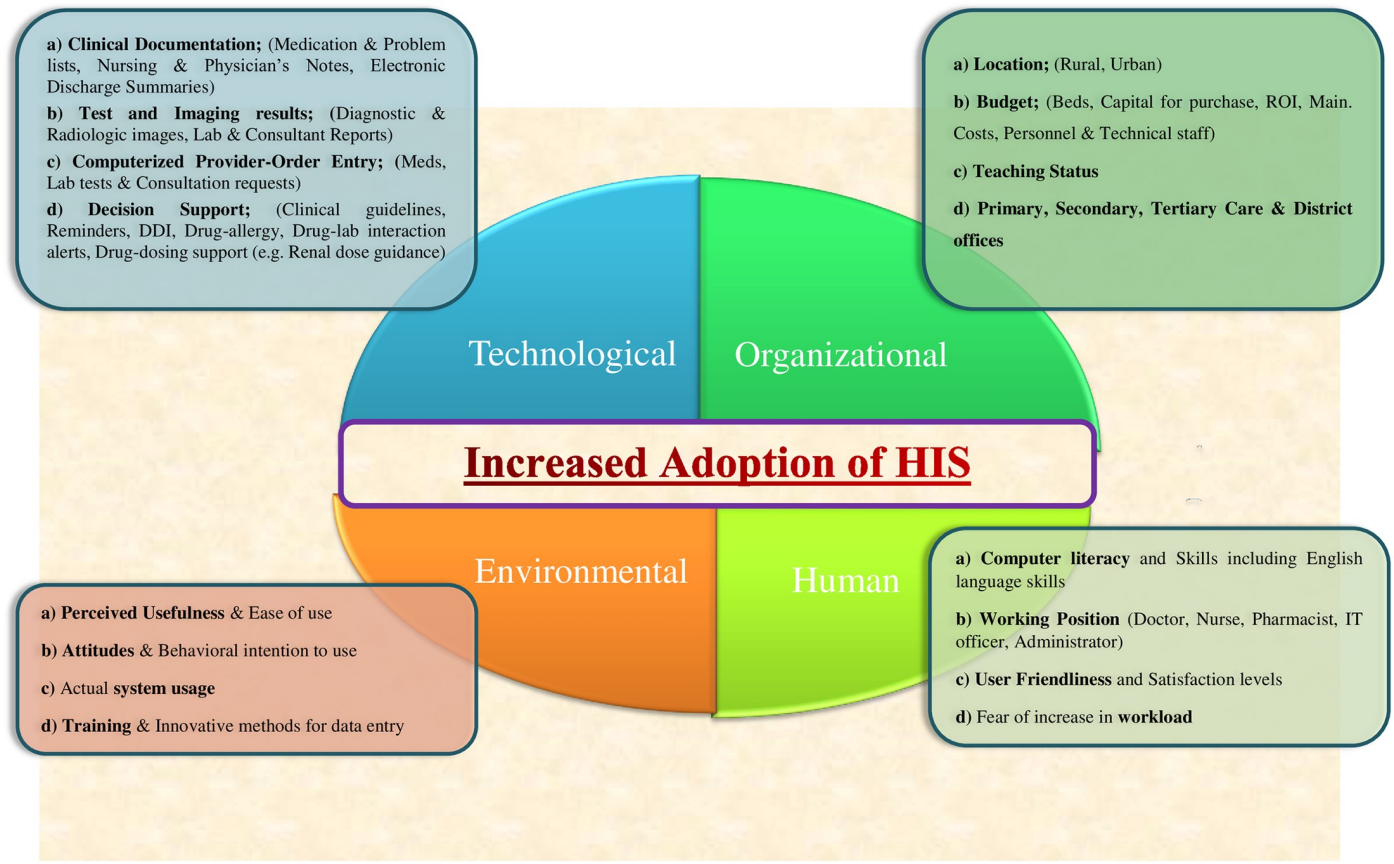
Conceptual framework of the study. A conceptual framework illustrating the relationship between technological, environmental, organizational and human factors regarding the successful adoption of health information systems.

The present study was designed to assess the health information system in Rawalpindi district and Islamabad Capital Territory, Pakistan with respect to technological, environmental and organizational factors affecting the adoption of health information management systems at district and sub-district level as well as tertiary, secondary and primary healthcare setups of Rawalpindi and Islamabad. Moreover, the study also explored the factors that served as barriers and constraints influencing the management and implementation of health information systems and also evaluated the perceptions of managers, heads and other healthcare professionals regarding the process factors of health information management systems at various health care levels. The study also closely examined the information and communication technologies infrastructure as well as provided solutions for improvement and management of the current health information system. The present study provides evidence-based data for policymakers to design and implement effective health information systems befitting to the local context of the country. The conceptual framework of the study is given (Fig 1).

## Research context

Pakistan is an emerging country with a population of over 200 million and has the potential to be among the top 20 economies of the world in the next 20 years [27]. However, the government has been spending a low percentage of its gross domestic product (GDP) on health for the last decade which is evident from the poor health infrastructure of the country. Government spending on health accounts for only 0.9% of the GDP however, at least 6% of the spending of GDP on health is recommended by WHO. There is no specific budget allocated for adoption of health information system, however, development of provincial HRH strategic frameworks, establishment of coherent HRH information system and a registry along with improving health information systems and upgrading software to DHIS2 to ensure timely, accurate and updated information for encouraging operational research to support decision-making, planning and monitoring processes have been added as health priorities in National Vision (2016–2025) of Pakistan [29]. A study conducted in “Shaukat Khanum Cancer Hospital” focused on the importance of electronic health and medical recording system reported that these systems had the potential in improving quality and therapeutic outcomes for the patient as well as in reducing errors related to medications [14]. Another study explored issues and prospects about e-health in Pakistan reported that for policymakers to make rational and judicious decisions across various healthcare systems operational in the country, hurdles encompassing scarce resource allocation, poor e-healthcare design, issues related to risk-benefit and cost analysis and socio-cultural barriers need to be resolved [30]. One major misconception about the electronic health technology systems could be the high capital cost associated with their development and implementation which might be the reason for their slow and inconsistent adoption worldwide. But the findings of the study focused on developing in-house health management and information system in a tertiary level hospital in Pakistan concluded the net savings of the project were 3.5 million USD with a payback duration of 3.4 years [14]. Moreover, a study exploring the underlying technological factors and ethical dimensions of digital health interventions carried out at the national level during the last five years highlighted the need to improve the outcomes of digital health initiatives by: (a) acquiring open-source software rather than patented versions, (b) refining current clinical practices by implementing updated digital tools and apps, (c) eliminating communication gap between various stakeholders and policymakers, (d) increased involvement and diligence by clinical experts and practicing physicians with the technology experts during planning, developing, and testing of such apps and devices, and (e) delineating regulatory frameworks and approvals required [31]. However, simply buying and implementing e-health technology-based systems without designing the proper framework, users’ readiness and preparedness to use these systems and addressing underlying organizational, environmental and socio-political issues would be unadvisable and would yield no effective clinical outcomes [30].

Pakistan, is the 5^th^ most populous country and having the potential to be in the top economies of the world, there surely is a need to develop and implement a health information management system (HIMS) at the national level for data collection, compilation, storing and analyzing information and transmission of data to provide feedback on all health-related information for policymakers in making accurate and timely decisions. A study conducted on the situation analysis of health management systems in Pakistan reported that although HIMS is generating information and its coverage is encouraging at present, but simultaneously it needs to be strengthened at various levels as it seems more ’data driven’ than ’action oriented’. Lack of coordination and duplication has been observed among various vertical health information systems along with time lag with respect to receiving of information and its dissemination. As geographic information system is comparatively a new concept in health sector in Pakistan due to which its application is inadequate. Better coordination among various vertical health information systems is required for strengthening of the whole system to practically contribute towards better decision-makings as well as to save resources [32].

Irrespective of the fact that implementing and regularizing the use of health information technologies in hospitals and institutions must result in more efficient and improved quality of care and health-related quality of life (HRQoL) for the patients, there are still no definitive estimates of the pervasiveness of adoption of the health information management systems in the healthcare facilities of Pakistan. Limited data is available on assessment of health information system in Pakistan while the available studies have mostly reviewed the existing literature or interventions in the field of digital health. Therefore, the present study was designed to assess the health information system (HIS) in terms technological, environmental and organizational factors affecting the adoption of health information management systems in Pakistan in order to provide evidence-based data for policymakers to design and implement effective health information systems befitting to the local context of the country.

## Methodology

**Fig 2:**
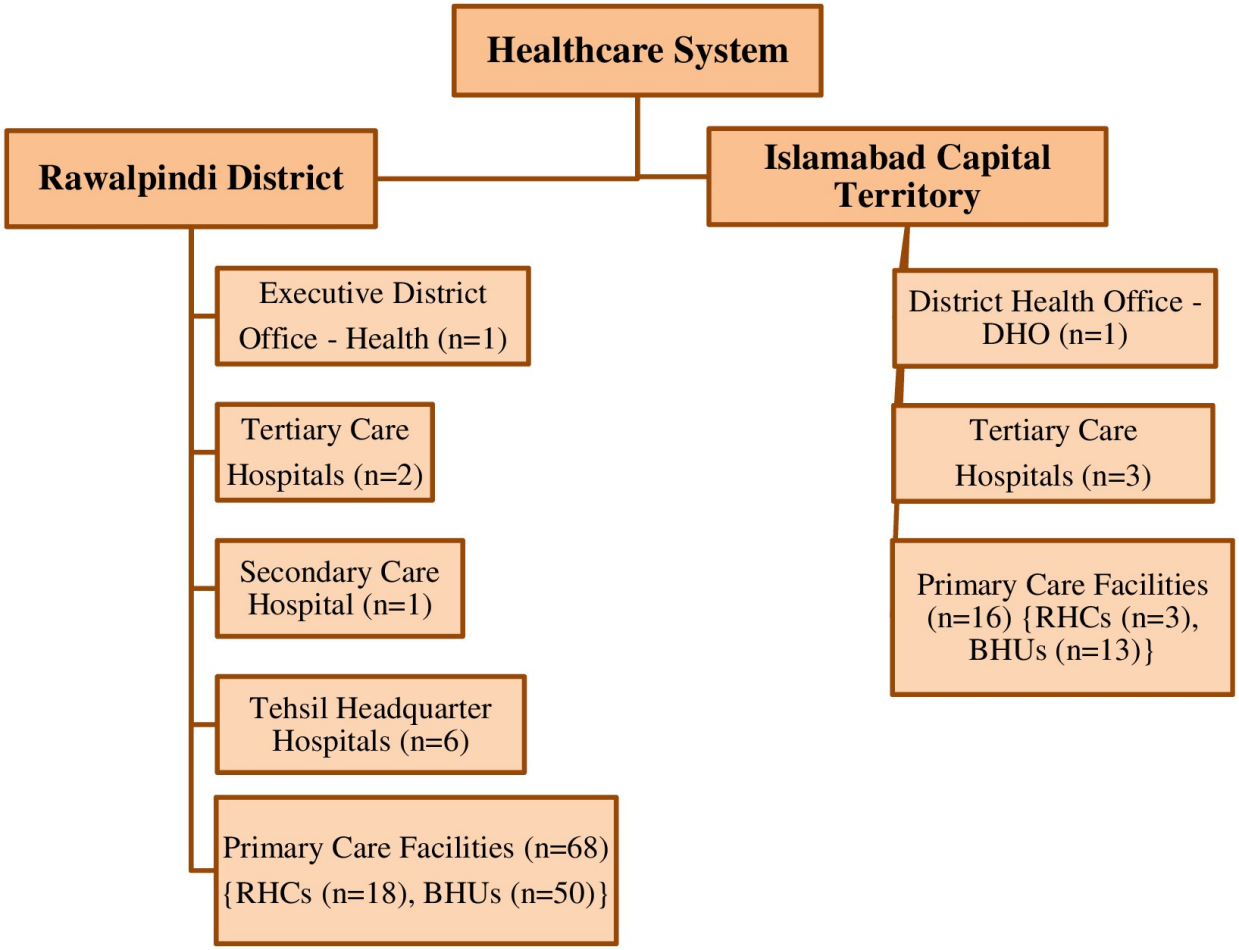
Sampling procedure and sample size. Out of a total of 97 healthcare facilities in both cities, 55 operational healthcare facilities were included in this study. Out of these 55 healthcare facilities, 10 were located in Islamabad while 45 were situated in Rawalpindi district. The healthcare facilities included in this study from different healthcare levels were: Tertiary healthcare (n = 5), secondary healthcare (n = 7) and primary healthcare (n = 41).

A descriptive cross-sectional study design was used to assess the health information system (HIS) in Rawalpindi district and Islamabad Capital Territory, Pakistan. Islamabad, being the capital city of Pakistan has always been the center point for various healthcare initiatives and the present government had also announced ‘Digital Pakistan Vision’ in 2019 with the prime focus to improve digital infrastructure, fostering innovation and entrepreneurship skills particularly in the field of IT and Health through various e-government programs as part of ‘Digital Transformation starting from the Capital.’ For this reason, ICT and Rawalpindi district being its twin city were chosen for healthcare information management system analysis. Furthermore, the idea behind choosing these was that as most of the budget is allocated to the capital city and the district from the most populous province Punjab, so whether the HIS in these privileged areas is up to the mark which could help to further visualize the situation in other comparatively unprivileged areas of the country. Study approval was taken from the Ethical Committee of Hamdard University (Ref. No. BASR-78-5). Approval was also taken from respective authorities (EDO-Health, District health officers, MS/CEO of hospitals and department heads) of different institutions from where data was collected. Besides this, consent was also taken from the respondents and their confidentiality of information was also ensured. Pre-validated data collection tools were used. Pre-validated tools used by the Department of Health, South Africa and Northwest University, South Africa for assessment of HIS of South Africa were used after prior approval from them. The healthcare systems of both countries are quite similar, yet the tools were slightly modified to fit in the local context in terms of facilities and healthcare budget allocation. The list of all the healthcare facilities within the jurisdiction of Rawalpindi District and Islamabad Capital Territory was obtained from respective district offices of health. Study sites for this research included all the primary, secondary and tertiary healthcare facilities of Rawalpindi District and Islamabad Capital Territory along with the District Health Office of Islamabad and District and Sub-District Health Offices of Rawalpindi, Pakistan. All the healthcare facilities in both cities were included in the study for the purpose of data collection except those facilities which were not operational. The sampling frame was comprised of professionally qualified physicians, pharmacists, nurses, admin and IT staff who directly interact and manage health information systems in hospitals and district as well as sub-district offices. Illustration of the sampling procedure along with sample size is shown in (Fig 2).

Prospective data were collected from June to December 2019 from primary sources by self-administering the questionnaires and getting them filled by the respondents on spot. Respondents belonging to different fields were approached by visiting them at their respective offices. After data collection, data was cleaned, coded and entered in SPSS version 21. Descriptive statistics comprising of frequency and percentages were calculated.

## Results

Of the total 71 facilities, 84.5% (n = 60) were located in Rawalpindi district and 15.5% (n = 11) were situated in Islamabad. Out of 60 facilities of Rawalpindi district, 85% (n = 51) were located in Rawalpindi, 6.67% (n = 4) in Taxila and 1.67% (n = 1) each in Gujjar Khan, Murree, Kahuta, Kotli Satiyaan and Kallar Sayedaan. Out of 71 respondents, 54.9% (n = 39) were from district office, 5.6% (n = 4) from sub-district office, 23.9% (n = 17) from hospital sector, while 14.1% (n = 10) were from rural health center and 1.4% (n = 1) from basic health unit. Of the total 71 respondents, 74.6% (n = 53) were male and 25.4% (n = 18) were female. A detailed description is given (Table 1).

**Table 1:** Demographic characteristics.

Indicator		n (%)
District	Rawalpindi	60 (84.5)
	Islamabad	11 (15.5)
Sub-District	Rawalpindi	51 (71.8)
	Gujjar Khan	1 (1.4)
	Taxila	4 (5.6)
	Murree	1 (1.4)
	Kahuta	1 (1.4)
	Kotli Satiyaan	1 (1.4)
	Kallar Sayedaan	1 (1.4)
	Islamabad	11 (15.5)
Work Environment	District Office	39 (54.9)
	Sub-District Office	4 (5.6)
	Hospital	17 (23.9)
	Rural Health Center (RHC)	10 (14.1)
	Basic Health Unit (BHU)	1 (1.4)
Age Category	More than 18 years—Less than 24 years	4 (5.6)
	More than 24 years—Less than 34 years	26 (36.6)
	More than 34 years—Less than 44 years	23 (32.4)
	More than 44 years—Less than 54 years	7 (9.9)
	More than 54 year—Less than 65 years	11 (15.5)
Gender	Male	53 (74.6)
	Female	18 (25.4)
Education	Diploma	5 (7.0)
	Bachelor’s Degree	19 (26.8)
	Master’s Degree	43 (60.6)
	PhD	4 (5.6)
Experience	Less than 1 year	3 (4.2)
	2 years to 5 years	28 (39.4)
	6 years to 10 years	10 (14.1)
	More than 10 years	30 (42.3)
Current Position	Executive District Officer (EDO) Health	3 (4.2)
	District Officer (Health)	3 (4.2)
	DHO Medical Services	1 (1.4)
	Deputy District Health Officer	2 (2.8)
	Assistant District Health Officer	3 (4.2)
	Drug Controller	1 (1.4)
	Deputy Drug Controller	7 (9.9)
	Provincial / District Quality Control Board	2 (2.8)
	MS DHQ Hospital	1 (1.4)
	Data-capturer in district office	1 (1.4)
	Administrative officer in district office	2 (2.8)
	Information technology head in district office	1 (1.4)
	Sub-district Health Information Officer	5 (7.0)
	Administrative officer in sub-district office	2 (2.8)
	Pharmacist/ Analyst at Drug Testing Laboratory	14 (19.7)
	Program Manager	1 (1.4)
	Administrative Officer in PHC	1 (1.4)
	CEO/ MS of hospital	3 (4.2)
	Clinical Head / Unit Head	2 (2.8)
	Data Capturer in hospital	1 (1.4)
	Information technology head in hospital	3 (4.2)
	Technical Director at Drug Testing Laboratory	1 (1.4)
	Additional Director at Drug Testing Laboratory	2 (2.8)
	Healthcare Provider in Primary Healthcare	9 (12.7)

The results of the present study reported that the resources available for the management of health information system included: Desk PCs (n = 70, 98.6%), laptops (n = 38, 53.5%), servers at the national or provincial level (n = 8, 11.3%) and tools for backup of data (n = 22, 31%). Access to Email was good, 81.7% (n = 58) of the respondents reported they had access to email, while only 25.4% (n = 18) had access to Intranet services. Around, 49.3% (n = 35) of the respondents, reported that they had no system in place to perform their responsibilities regarding the management of health information data. A detailed description is given (Table 2).

**Table 2:** Availability of resources for management of HIS.

Indicator	Yes	No
	n (%)	n (%)
Desk PCs available to support responsibilities regarding the management of Health Information Data.	70 (98.6)	1 (1.4)
Laptop/ Notebook PCs available to support responsibilities regarding the management of Health Information Data.	38 (53.5)	33 (46.5)
Servers at National and Provincial level available to support responsibilities regarding the management of Health Information Data.	8 (11.3)	63 (88.7)
Tools for backup of data (software or hardware) available to support responsibilities regarding the management of Health Information Data.	22 (31.0)	49 (69.0)
Black and white printers available to support responsibilities regarding the management of Health Information Data.	58 (81.7)	13 (18.3)
Color printers available to support responsibilities regarding the management of Health Information Data.	30 (42.3)	41 (57.7)
Scanners available to support responsibilities regarding the management of Health Information Data.	38 (53.5)	33 (46.5)
Digital Projectors available to support responsibilities regarding the management of Health Information Data.	29 (40.8)	42 (59.2)
Photocopiers available to support responsibilities regarding the management of Health Information Data.	40 (56.3)	31 (43.7)
Do you have access to E-mail?	58 (81.7)	13 (18.3)
Do you have access to Intranet?	18 (25.4)	53 (74.6)
Do you have access to Internet?	60 (84.5)	11 (15.5)
How reliable are the E-mail services?	Very Unreliable	13 (18.3)
	Unreliable	2 (2.8)
	Reliable	34 (47.9)
	Very Reliable	21 (29.6)
How reliable are the Intranet services?	Very Unreliable	40 (56.3)
	Unreliable	2 (2.8)
	Not Sure	12 (16.9)
	Reliable	14 (19.7)
	Very Reliable	3 (4.2)
How reliable are the Internet services?	Very Unreliable	11 (15.5)
	Unreliable	4 (5.6)
	Not Sure	6 (8.5)
	Reliable	35 (49.3)
	Very Reliable	14 (19.7)
Percentage of time spent on health information data management (daily usage)?	More than 75%– 100%	7 (9.9)
	More than 50% Less than 75%	8 (11.3)
	More than 25% Less than 50%	7 (9.9)
	Less than 25%	14 (19.7)
	System does not exist	35 (49.3)

The results of the study reported that 45.1% (n = 32) of the respondents answered their computer literacy was good while 42.3% (n = 30) reported an average computer literacy. Most of the respondents, 85.9% (n = 61) answered that they had not received any sort of training related to the management of health information system related activities (Table 3).

**Table 3:** Training on management of health information system.

**Indicator**	**n (%)**	
How would you rate your computer literacy?	Poor	2 (2.8)
	Average	30 (42.3)
	Good	32 (45.1)
	Excellent	7 (9.9)
**Training**	**Yes**	**No**
	**n (%)**	**n (%)**
Have you received any training in HIS related activities in the last six months?	10 (14.1)	61 (85.9)
Do you think you’re adequately trained in health information data collection?	40 (56.3)	31 (43.7)
Do you think you’re adequately trained in health information data capturing and storage?	35 (49.3)	36 (50.7)
Do you think you’re adequately trained in health information data transmission?	31 (43.7)	40 (56.3)
Do you think you’re adequately trained in health information data collation?	16 (22.5)	55 (77.5)
Do you think you’re adequately trained in health information data analysis?	18 (25.4)	53 (74.6)
Do you think you’re adequately trained in health information data reporting and provision of feedback?	29 (40.8)	42 (59.2)
Do you think you’re adequately trained in health information data use?	15 (21.1)	56 (78.9)
Do you think you’re adequately trained in health information data quality assessment?	11 (15.5)	60 (84.5)

Of the total 71 respondents, 28.2% (n = 20) agreed that the decisions were based on the personnel liking of the superiors while 36% (n = 50.7) agreed that the decisions were based on evidence and facts/statistics. On the other hand, 29.6% (n = 21) of the respondents agreed that decisions were based on the health information system data whereas 21.1% (n = 15) stated that decisions were based on the political interference (Table 4).

**Table 4:** Decision making regarding management of health information system.

Indicator	n (%)	
How strongly you agree that in your health facility, decisions are based on the personnel liking of the personnel?	Strongly disagree	6 (8.5)
	Somewhat disagree	2 (2.8)
	Disagree	13 (18.3)
	Neither disagree or agree	21 (29.6)
	Somewhat agree	20 (28.2)
	Agree	8 (11.3)
	Strongly agree	1 (1.4)
How strongly you agree that in your health facility, decisions are based on the superior’s directive?	Strongly disagree	2 (2.8)
	Somewhat disagree	1 (1.4)
	Disagree	4 (5.6)
	Neither disagree or agree	14 (19.7)
	Somewhat agree	31 (43.7)
	Agree	14 (19.7)
	Strongly agree	3 (4.2)
How strongly you agree that in your health facility, decisions are based on the evidence, facts or statistics?	Strongly disagree	2 (2.8)
	Disagree	1 (1.4)
	Neither disagree or agree	7 (9.9)
	Somewhat agree	16 (22.5)
	Agree	36 (50.7)
	Strongly agree	9 (12.7)
How strongly you agree that in your health facility, decisions are based on the political interference?	Strongly disagree	7 (9.9)
	Disagree	8 (11.3)
	Neither disagree or agree	12 (16.9)
	Somewhat agree	11 (15.5)
	Agree	15 (21.1)
	Strongly agree	18 (25.4)
How strongly you agree that in your health facility, decisions are based on comparing data with strategic health objectives?	Strongly disagree	1 (1.4)
	Neither disagree or agree	11 (15.5)
	Somewhat agree	26 (36.6)
	Agree	25 (35.2)
	Strongly agree	8 (11.3)
How strongly you agree that in your health facility, decisions are based on considering costs?	Neither disagree or agree	14 (19.7)
	Somewhat agree	15 (21.1)
	Agree	35 (49.3)
	Strongly agree	7 (9.9)
How strongly you agree that in your health facility, decisions are based on the community health needs?	Neither disagree or agree	8 (11.3)
	Somewhat agree	15 (21.1)
	Agree	40 (56.3)
	Strongly agree	8 (11.3)
How strongly you agree that in your health facility, decisions are based on the health information system data?	Neither disagree or agree	31 (43.7)
	Somewhat agree	13 (18.3)
	Agree	21 (29.6)
	Strongly agree	6 (8.5)

Of the total 71 respondents, the majority of them 54.9% (n = 39) responded that the superiors promote teamwork, while 39.4% (n = 28) of the respondents agreed that superiors were open to alternative views. The majority of the respondents 29.6% (n = 21) answered that the personnel were not punctual regarding documentation of health information system data (Table 5).

**Table 5:** Leadership capacity regarding management of health information system.

Indicator	n (%)	
In your health facility, superiors promote teamwork?	Neither disagree or agree	1 (1.4)
	Somewhat agree	19 (26.8)
	Agree	39 (54.9)
	Strongly agree	12 (16.9)
In your health facility, superiors are open to alternative views?	Somewhat disagree	1 (1.4)
	Disagree	13 (18.3)
	Neither disagree or agree	16 (22.5)
	Somewhat agree	28 (39.4)
	Agree	12 (16.9)
	Strongly agree	1 (1.4)
In your health facility, superiors listen to employees’ ideas and concerns?	Somewhat disagree	2 (2.8)
	Disagree	11 (15.5)
	Neither disagree or agree	18 (25.4)
	Somewhat agree	30 (42.3)
	Agree	9 (12.7)
	Strongly agree	1 (1.4)
In your health facility, superiors allow disagreements before reaching a decision?	Strongly disagree	1 (1.4)
	Somewhat disagree	1 (1.4)
	Disagree	33 (46.5)
	Neither disagree or agree	14 (19.7)
	Somewhat agree	13 (18.3)
	Agree	9 (12.7)
In your health facility, superiors are concerned about serving target communities’ needs?	Disagree	5 (7.0)
	Neither disagree or agree	6 (8.5)
	Somewhat agree	35 (49.3)
	Agree	22 (31.0)
	Strongly agree	3 (4.2)
In your health facility, superiors seek feedback from concerned persons working with health information data?	Somewhat disagree	2 (2.8)
	Disagree	17 (23.9)
	Neither disagree or agree	13 (18.3)
	Somewhat agree	28 (39.4)
	Agree	11 (15.5)
In your health facility, superiors discuss conflicts openly to resolve them?	Strongly disagree	2 (2.8)
	Somewhat disagree	4 (5.6)
	Disagree	38 (53.5)
	Neither disagree or agree	11 (15.5)
	Somewhat agree	8 (11.3)
	Agree	8 (11.3)
In your health facility, superiors use health information data for setting of targets?	Somewhat disagree	1 (1.4)
	Disagree	21 (29.6)
	Neither disagree or agree	15 (21.1)
	Somewhat agree	20 (28.2)
	Agree	12 (16.9)
	Strongly agree	2 (2.8)
In your health facility, superiors check health information data quality?	Somewhat disagree	1 (1.4)
	Disagree	20 (28.2)
	Neither disagree or agree	15 (21.1)
	Somewhat agree	24 (33.8)
	Agree	11(15.5)
In your health facility, superiors provide regular feedback to staff?	Somewhat disagree	2 (2.8)
	Disagree	13 (18.3)
	Neither disagree or agree	21 (29.6)
	Somewhat agree	26 (36.6)
	Agree	9 (12.7)

Of the total 71 respondents, 52.1% (n = 37) agreed that the personnel performed their duties honestly and were punctual regarding documentation of health information data. Moreover, 21.1% (n = 15) of the respondents agreed that personnel could gather HIS data to find the root causes of the problem whereas 19.7% (n = 14) stated that personnel use HIS data for day-to-day management of the facility. Furthermore, 22.5% (n = 16) stated that personnel were made accountable for poor performance. A detailed description is given (Table 6).

**Table 6:** Personnel capacity regarding management of health information system.

Indicator	n (%)	
In your health facility, personnel perform duties honestly?	Disagree	2 (2.8)
	Neither disagree or agree	3 (4.2)
	Somewhat agree	27 (38.0)
	Agree	37 (52.1)
	Strongly agree	2 (2.8)
In your health facility, personnel are punctual regarding documentation of health information data?	Somewhat disagree	1 (1.4)
	Disagree	21 (29.6)
	Neither disagree or agree	10 (14.1)
	Somewhat agree	19 (26.8)
	Agree	18 (25.4)
	Strongly agree	2 (2.8)
In your health facility, personnel help each other in serving the patients / communities	Disagree	8 (11.3)
	Neither disagree or agree	2 (2.8)
	Somewhat agree	31 (43.7)
	Agree	29 (40.8)
	Strongly agree	1 (1.4)
In your health facility, personnel set appropriate and doable health status targets.	Disagree	10 (14.1)
	Neither disagree or agree	9 (12.7)
	Somewhat agree	31 (43.7)
	Agree	19 (26.8)
	Strongly agree	2 (2.8)
In your health facility, personnel feel guilty for not accomplishing the set target / performance.	Disagree	10 (14.1)
	Neither disagree or agree	11 (15.5)
	Somewhat agree	31 (43.7)
	Agree	14 (19.7)
	Strongly agree	5 (7.0)
In your health facility, personnel use HIS data for day-to-day management of the facility.	Disagree	27 (38.0)
	Neither disagree or agree	12 (16.9)
	Somewhat agree	14 (19.7)
	Agree	14 (19.7)
	Strongly agree	4 (5.6)
In your health facility, personnel can gather HIS data to find the root cause(s) of the problem.	Somewhat disagree	1 (1.4)
	Disagree	16 (22.5)
	Neither disagree or agree	21 (29.6)
	Somewhat agree	17 (23.9)
	Agree	15 (21.1)
	Strongly agree	1 (1.4)
In your health facility, personnel can develop appropriate outcomes for a particular intervention.	Somewhat disagree	2 (2.8)
	Disagree	12 (16.9)
	Neither disagree or agree	21 (29.6)
	Somewhat agree	22 (31.0)
	Agree	13 (18.3)
	Strongly agree	1 (1.4)
In your health facility, personnel are able to say no to colleagues for demands/ decisions not supported by evidence.	Strongly disagree	4 (5.6)
	Somewhat disagree	3 (4.2)
	Disagree	24 (33.8)
	Neither disagree or agree	16 (22.5)
	Somewhat agree	14 (19.7)
	Agree	9 (12.7)
	Strongly agree	1 (1.4)
In your health facility, personnel are made accountable for poor performance.	Disagree	5 (7.0)
	Neither disagree or agree	15 (21.1)
	Somewhat agree	34 (47.9)
	Agree	16 (22.5)
	Strongly agree	1 (1.4)

Of the total 71 respondents, 36.6% (n = 26) strongly agreed that collecting health information data was useful and 38% (n = 27) agreed that collecting health information data was meaningful for them. The majority of the respondents 54.9% (n = 39) agreed that data was required to monitor facility performance (Table 7).

**Table 7:** Personal feelings regarding health information data.

Indicator	n (%)	
Do you feel collecting health information data is useful?	Neither disagree or agree	9 (12.7)
	Somewhat agree	14 (19.7)
	Agree	22 (31.0)
	Strongly agree	26 (36.6)
Do you feel that collecting health information data that is not used for decision making discourages you?	Disagree	8 (11.3)
	Neither disagree or agree	19 (26.8)
	Somewhat agree	18 (25.4)
	Agree	18 (25.4)
	Strongly agree	8 (11.3)
Do you feel that collecting health information data makes you feel bored?	Strongly Disagree	3 (4.2)
	Somewhat disagree	8 (11.3)
	Disagree	22 (31.0)
	Neither disagree or agree	30 (42.3)
	Somewhat agree	3 (4.2)
	Agree	5 (7.0)
Do you feel that collecting health information data is meaningful for you?	Strongly Disagree	1 (1.4)
	Somewhat disagree	1 (1.4)
	Disagree	8 (11.3)
	Neither disagree or agree	11 (15.5)
	Somewhat agree	19 (26.8)
	Agree	27 (38.0)
	Strongly agree	4 (5.6)
Do you feel that collecting health information data gives you the feeling that data is needed to monitor facility performance?	Disagree	3 (4.2)
	Neither disagree or agree	6 (8.5)
	Somewhat agree	15 (21.1)
	Agree	39 (54.9)
	Strongly agree	8 (11.3)
Do you feel that collecting health information data gives you the feeling that it is forced on you?	Strongly Disagree	3 (4.2)
	Somewhat disagree	8 (11.3)
	Disagree	23 (32.4)
	Neither disagree or agree	28 (39.4)
	Somewhat agree	4 (5.6)
	Agree	5 (7.0)
Do you feel that collecting health information data is appreciated by co-workers and superiors?	Somewhat disagree	2 (2.8)
	Disagree	9 (12.7)
	Neither disagree or agree	23 (32.4)
	Somewhat agree	20 (28.2)
	Agree	16 (22.5)
	Strongly agree	1 (1.4)
Do you feel that health information data collecting and reporting take too much of your time?	Strongly Disagree	2 (2.8)
	Disagree	7 (9.9)
	Neither disagree or agree	22 (31.0)
	Somewhat agree	19 (26.8)
	Agree	21 (29.6)

Of the total 71 respondents, the majority of them 33.8% (n = 24) agreed that health information data in their facility was used to optimize patient care and public health. While 38% (n = 27) of the respondents agreed that health information data was used to optimize the overall health status of the population (Table 8).

**Table 8:** Purpose of health information data.

Indicator	n (%)	
In your facility/office, health information data is used to optimize patient care.	Disagree	10 (14.1)
	Neither disagree or agree	8 (11.3)
	Somewhat agree	17 (23.9)
	Agree	24 (33.8)
	Strongly agree	12 (16.9)
In your facility/office, health information data is used to optimize public health.	Disagree	10 (14.1)
	Neither disagree or agree	7 (9.9)
	Somewhat agree	18 (25.4)
	Agree	24 (33.8)
	Strongly agree	12 (16.9)
In your facility/office, health information data is used to optimize the health status of the population.	Disagree	10 (14.1)
	Neither disagree or agree	7 (9.9)
	Somewhat agree	17 (23.9)
	Agree	27 (38.0)
	Strongly agree	10 (14.1)
In your facility/office, health information data is used to optimize performance of health programmes.	Disagree	15 (21.1)
	Neither disagree or agree	8 (11.3)
	Somewhat agree	13 (18.3)
	Agree	21 (29.6)
	Strongly agree	14 (19.7)
In your facility/office, health information data is used to monitor, evaluate and report on performance against all legislated plans in the health sector.	Disagree	15 (21.1)
	Neither disagree or agree	9 (12.7)
	Somewhat agree	12 (16.9)
	Agree	24 (33.8)
	Strongly agree	11 (15.5)
In your facility/office, health information data is used to collect data for Morbidity and Mortality based quantification.	Disagree	10 (14.1)
	Neither disagree or agree	8 (11.3)
	Somewhat agree	19 (26.8)
	Agree	22 (31.0)
	Strongly agree	12 (16.9)

Out of the total 71 respondents, only 14.1% (n = 10) partially agreed that they could check data accuracy of health information system related activities. Only 12.7% (n = 9) of the respondents partially agreed that they were able to compute trends (Table 9).

**Table 9:** Self-efficacy for performing tasks related to health information systems.

Indicator	n (%)	
How confidently you can check data accuracy of different HIS activities?	Zero Percent	16 (22.5)
	≤ 25%	20 (28.2)
	> 25%–≤ 50%	21 (29.6)
	> 50%–≤ 75%	10 (14.1)
	> 75% to 100%	4 (5.6)
How confidently you can calculate percentages correctly of different HIS activities?	Zero Percent	17 (23.9)
	≤ 25%	17 (23.9)
	>25%–≤ 50%	19 (26.8)
	>50%–≤ 75%	14 (19.7)
	>75% to 100%	4 (5.6)
How confidently you can compute trends from charts of different HIS activities?	Zero Percent	19 (26.8)
	≤ 25%	18 (25.4)
	>25%–≤ 50%	21 (29.6)
	>50%–≤ 75%	9 (12.7)
	>75% to 100%	4 (5.6)
How confidently you can plot data by months or years?	Zero Percent	19 (26.8)
	≤ 25%	18 (25.4)
	>25% -≤ 50%	19 (26.8)
	>50%–≤ 75%	11 (15.5)
	>75% to 100%	4 (5.6)
How confidently you can explain findings of different HIS activities?	Zero Percent	25 (35.2)
	≤ 25%	23 (32.4)
	>25%–≤ 50%	9 (12.7)
	>50%–≤ 75%	12 (16.9)
	>75% to 100%	2 (2.8)
How confidently you can explain the implications of findings or results of different HIS activities?	Zero Percent	30 (42.3)
	≤ 25%	21 (29.6)
	>25%–≤ 50%	7 (9.9)
	> 50%–≤ 75%	13 (18.3)
How confidently you can use data to identify gaps of different HIS activities?	Zero Percent	37 (52.1)
	≤ 25%	14 (19.7)
	> 25%–≤ 50%	11 (15.5)
	>50%–≤ 75%	7 (9.9)
	> 75% to 100%	2 (2.8)
How confidently you can use data to set targets of different HIS activities?	Zero Percent	33 (46.5)
	≤ 25%	16 (22.5)
	> 25%–≤ 50%	11 (15.5)
	> 50%–≤ 75%	9 (12.7)
	> 75% to 100%	2 (2.8)
How confidently you can use data to make various types of decisions and provide feedback?	Zero Percent	32 (45.1)
	≤ 25%	14 (19.7)
	> 25%–≤ 50%	13 (18.3)
	> 50%–≤ 75%	10 (14.1)
	> 75% to 100%	2 (2.8)

A comparison regarding the process factors of health information system among different healthcare levels revealed that availability of equipment and services in all the healthcare facilities was shown to be moderate. However, majority of the respondents at primary healthcare level were unsatisfied with the equipment and the available services. Computer literacy was average among all the respondents. Human resources and training were inadequate at tertiary, secondary and primary healthcare levels. Data was mostly stored manually apart from the district office, where health information system was slightly used for data storage and analysis. Utilization of the data obtained from health information system and feedback on the submitted data was shown to be inadequate at the hospitals and sub-district level. Health information system was observed to be partially functional at district and sub-district level while completely unavailable at tertiary, secondary and primary healthcare levels (Table 10).

**Table 10:** Comparison of health information system among different healthcare levels.

Indicators	District Office	Sub-District Office	Hospitals (Tertiary & Secondary Care)	Primary Healthcare (RHCs & BHUs)	Inferences
Equipment (Desk PCs, Laptops, Servers etc.)	Moderately Available	Moderately Available	Moderately Available	Moderately Unavailable	Largely because of limited resources and budget allocation
Services (Email, Intranet and Internet)	Sufficient	Sufficient	Sufficient	Insufficient	Largely because of limited resources and budget allocation
Satisfaction with the Equipment and Services	Satisfied	Satisfied	Partially satisfied	Unsatisfied	Mostly outdated models were used at Primary Healthcare
Electricity Interruption	Occasionally	Daily	Daily	Daily	No availability of back-up generators
Computer Literacy	Good	Average	Average	Average	IT staff was insufficient and mostly absent in most of the facilities
Human Resources and Workforce	Sufficient	Moderately sufficient	Insufficient	Insufficient	No schedule of regular recruitments and periodic trainings, which largely depicts the inefficiency of policy makers
Training in HIS related activities (data collection, analysis, collation, transmission, reporting etc.)	Moderately trained	Inadequately trained	Inadequately trained	Inadequately trained	No schedule of regular recruitments and periodic trainings, which largely depicts the inefficiency of policy makers
Availability of priority documents (National Health Act, District and Provincial etc.)	Partially Available	Unavailable	Partially Available	Unavailable	Poor planning and resource allocation processes leading to low compliance of SOPs.
% of time spent on HIS management	25–50%	< 25%	< 25%	< 25%	Either the staff was untrained on how to use the health information system or the system was not in place.
Data Collection, Storage and Analysis	Mostly manual, slightly on HIS	Both Manual and HIS	Manual	Manual	Either the staff was untrained on how to use the health information system or the system was not in place.
Utilization of HIS data	Adequate	Inadequate	Inadequate	Inadequate	Either the staff was untrained on how to use the health information system or the system was not in place.
Constraints in HIS data management (administrative, HR, Financial etc.)	Moderate	Moderate	Severe	Severe	Most of the meetings largely focused on different health programs (Polio, Dengue, MCHC, TB etc.) and less on Health Information System data to optimize patient care and health status of the population
Schedule of routine meetings for management of HIS data	Monthly	Monthly	No Schedule	No Schedule	Most of the meetings largely focused on different health programs (Polio, Dengue, MCHC, TB etc.) and less on Health Information System data to optimize patient care and health status of the population
Feedback on the data submitted to Provincial / District offices	Frequent	Seldom	Never	Never	Most of the meetings largely focused on different health programs (Polio, Dengue, MCHC, TB etc.) and less on Health Information System data to optimize patient care and health status of the population
Health Information System Exist	Partially Exist	Partially Exist	System does not exist	System does not exist	---------------------

## Conclusions and discussion

Health information management system is a vital tool for all the healthcare system building blocks guiding institutions for continuous monitoring and periodic evaluation in optimizing patient care and achieving better outcomes [7]. Any healthcare institution aims to provide high quality medical care to its patients. The results of the present study showed that the health information system in Islamabad and Rawalpindi district is not well established. The equipment (desk PCs, laptops, printers, etc.) were moderately available at district, sub-district, tertiary and secondary care levels while mostly unavailable at the primary healthcare setups. The staff at the primary healthcare levels also reported dissatisfaction with the available equipment and services as mostly outdated equipment were the part of the primary healthcare setups. Similar findings from a study conducted in Saudi Arabia also highlighted the unavailability of computers particularly laptop at different healthcare levels [3]. The results of the present study also highlighted administrative, financial and human resources as the most significant barriers towards health information data management. Similarly, the barriers related to behaviors and attitudes of the administration in the use of health information technology systems were also reported by another study conducted in Pakistan [24]. Moreover, lack of capital and unavailability of staff was highlighted as the major barriers towards successful adoption of HIS system in the USA [20].

Effective health information management systems at different healthcare levels can significantly decrease medical errors [5]. The results of the present study reported that the health information data is used to optimize patient care and health status of the population as well as to improve public health at the district and sub-district levels. Similar findings were reported from another study highlighting that the health information systems improved the quality and efficiency in achieving the desired health outcomes for the population in Canada [6]. The results of the present study also highlighted the availability of a moderately sufficient workforce in health information system related activities at district and sub-district levels, while an insufficient human resource at primary, secondary and tertiary care hospitals. Moreover, the staff working at these facilities was either inadequately trained in data management activities or the data was unavailable for those facilities where suboptimal training was conducted. These findings are in line with a study conducted in Indonesia which highlighted lack of training and skills in data management processes of health information systems [25]. Moreover, the results of the current study showed that the health information system partially exists at district and sub-district levels, while was completely absent at tertiary, secondary and primary healthcare levels. Most of the primary healthcare facilities were found to be non-operational. Scarce resources and limited budget allocation for health might also be associated with the low adoption of health information systems. The results of the present study are in line with another study conducted in Malaysia which reported that only 21 out of 138 public sector hospitals had implemented the health information system [21].

Health IT, particularly electronic medical records can improve the productivity, efficiency and effectiveness of health care professionals. However, stagnant adoption of these technologies in healthcare systems, difficulty in using or lacking technical skills to operate, systems not fully interoperable, technology and capacity in terms of infrastructure and resources are considered as major hindrance towards adoption of health information systems in Pakistan. Most of the studies conducted till date in Pakistan related to HIS has focused on assessment of medication errors, their associated causes or importance of electronic health and medical recording system in their prevention. However, the present study is the first of its kind to the best of our knowledge which provides a comprehensive analysis of current HIS in terms of technological, environmental, organizational and human factors affecting successful adoption of health information systems all together technical, organizational, behavioral and human factors influencing the adoption of health information management systems in Pakistan. The present study provides baseline data for the policymakers to design effective strategies for implementation of effective health information management systems on district and provincial level in the initial phase and later at the national level befitting to the local context of the country. The study also provides insight regarding the problems, challenges and barriers associated with the management of health information systems along with the information and communication technologies infrastructure along with user’s satisfaction with the current HIS. In addition, it also gives in-depth of the process factors of health information management systems at district and sub-district levels as well as tertiary, secondary and primary healthcare facilities in Islamabad and Rawalpindi District which needs to be improved for effective implementation of HIS in Pakistan.

The present study has been carried out in the Rawalpindi district and Islamabad capital territory. Thus, the results cannot be generalized to the whole country. Most of the facilities at the primary healthcare level were nonoperational. Moreover, the availability of managers, officers and staff and the willingness to participate in the study was another challenge faced during data collection. Scarcity of literature, particularly related to HIS of Pakistan was another major obstacle faced during conduction of the study. Time and financial constraints were a few of the other limitations of the study. Furthermore, few factors that affect the adoption of health information systems were identified but being not the part of the study objectives analysis was not performed to validate the associations among these factors which is another limitation of the current study.

The present study concluded that the health information system of Pakistan was not up to the mark. The health information management system partially existed at district and sub-district offices, while was completely absent at tertiary, secondary and primary healthcare levels. Even at district and sub-district offices, it was found that only the Basic Health Information System (BHIS) was in place. This can be attributed to the fact that, in the past, improving the public health of the population has not been a key mandate for the governments which is evident from the low budget spending on health. The poor adoption of health information technology systems at healthcare facilities can largely be attributed to insufficient human resources with no schedule of periodic recruitments and on-job training. Also, the equipment and services available at these health facilities were outdated and these institutions are still being run by outdated methods. Effective and timely strategies involving all the stakeholders and healthcare professionals must be designed and implemented at the National level to build an affordable, resilient and quality healthcare system through advancements in the field of information and communication technologies.

Future research involving both quantitative and qualitative approaches must be conducted to understand and resolve the issues related to the integration and use of health information management systems along with validation of association among these factors affecting the adoption of health information systems. Moreover, studies focusing on utilizing health information data to build patient-centric healthcare models suitable for the economic growth and prosperity of the country must be devised. Studies exploring effective strategies for implementation of the health information management systems at the national level might also prove to be conducive for equitable and resilient healthcare systems.

## Supporting information

S1 Questionnaire (SAV)

S2 Questionnaire (SAV)

S3 Questionnaire (SAV)

S4 Questionnaire (SAV)

S5 Questionnaire (SAV)

S6 Questionnaire (SAV)

S7 Questionnaire (SAV)
